# Bio-psycho-social model of aggressive behaviour

**DOI:** 10.1192/j.eurpsy.2022.1546

**Published:** 2022-09-01

**Authors:** A. Mihai

**Affiliations:** George Emil Palade” University of Medicine, Pharmacy, Science and Technology of Târgu Mureș,, M4, Psychiatry Department, Tg Mures, Romania

**Keywords:** Bio-psycho-social model, aggressive behavior, didactic model

## Abstract

**Introduction:**

The state of knowledge from scientific literature will be presented from biologic perspective, psychopathology and social context in development of agression.

**Objectives:**

The aim of this presentation is to create a bio-psycho-socio model of agression.

**Methods:**

The literature research in risk factors of aggressive behaviour was done, and the results grouped in three domains biologic, psychologic and social. A didactic bio-psycho-socio model was constructed.

**Results:**

The complex picture of aggressivity could be distorted if we reduce understanding to a narrow super-specialization perspective. This presentation enlarge approach with genetic, endocrine, neurologic, psychologic and sociologic perspective. All this data will be include in a schematic bio-psycho-socio model, and describe the application in mental health practice in understanding the patients with psychiatric disorder. The main result will be a Bio-psycho-social model of aggressive behavior, which could be helpful in understanding and predicting aggressive behavior.

**Conclusions:**

Complex perspective of aggresive behaviour could help better understand and prevent aggresive behaviour

**Disclosure:**

No significant relationships.

